# Cumulative, high-stress calls impacting adverse events among law enforcement and the public

**DOI:** 10.1186/s12889-020-09219-x

**Published:** 2020-07-20

**Authors:** Katelyn K. Jetelina, Alaina M. Beauchamp, Jennifer M. Reingle Gonzalez, Rebecca J. Molsberry, Stephen A. Bishopp, Simon Craddock Lee

**Affiliations:** 1 Department of Epidemiology, Human Genetics, and Environmental Sciences, University of Texas Health Science Center at Houston, School of Public Health, Dallas Regional Campus, 6011 Harry Hines Blvd, Dallas, TX USA; 2 Department of Population and Data Sciences, University of Texas Southwestern Medical Center, Dallas, TX USA; 3 Meadows Mental Health Policy Institute, Dallas, TX USA; 4 Dallas Police Department, Dallas, TX USA

## Abstract

**Background:**

The unpredictable, and sometimes dangerous, nature of the occupation exposes officers to both acute and chronic stress over law enforcement officers’ (LEO)  tenure. The purpose of this study is two-fold: 1) Describe multi-level characteristics that define high-stress calls for service for LEO; and 2) Characterize factors that impact cumulative stress over the course of a LEO’s shift.

**Methods:**

Qualitative data were collected from 28 LEOs at three law enforcement agencies in the Dallas-Fort Worth areas from April 2019 to February 2020. Focus group data were iteratively coded by four coders using inductive and deductive thematic identification.

**Results:**

Five multi-level factors influenced officer stress: 1) officer characteristics (e.g. military experience; gender); 2) civilian behavior (e.g. resistance, displaying a weapon); 3) supervisor factors (micromanagement); 4) environmental factors (e.g. time of year); and, 5) situational factors (e.g. audience present; complexity of calls). Four themes that characterized cumulative stress: 1) cyclical risk; 2) accelerators; 3) decelerators; and 4) experience of an adverse event.

**Conclusions:**

LEOs become susceptible to adverse events (e.g. injury, excessive use of force) after repeated exposure to high-stress calls for service. Ongoing exposures to stress continue to occur throughout the shift. Our long-term goal is to interrupt this repetitive, cumulative process by restricting the number of consecutive high-risk, high-intensity calls an officer is permitted to respond to.

## Background

Law enforcement officers (LEOs) are exposed to calls for service of a traumatic nature on a daily basis [1]. The unpredictable, and sometimes dangerous, nature of the occupation exposes officers to both acute (i.e. “flight or fight”) and chronic stress over a LEO’s tenure [2–4]. Over the past two decades, a plethora of literature has shown that stressors commonly experienced among law enforcement impact adverse physiological (e.g. heart rate, cortisol) [2, 5, 6], psychological (e.g. anxiety, depression, PTSD, burnout) [5, 7–11], and physical (e.g. chronic back pain, heart disease) [5, 7–11] outcomes.

Far fewer studies have identified factors that influence whether an officer perceives a call for service as “high-stress”. In a single day, officers respond to calls for service for a number of reasons, ranging from seemingly minor or low risk events (such as traffic accidents and noise complaints) to active shooters or incidents that may result in personal injury or death to the officer. Researchers have typically focused on how the classification of a call, such as family violence calls, entrapment calls, and suspicious persons or circumstances, influences stress [4, 12–14]. For example, Violanti and colleagues discovered that the most frequent LEO stressor was family disputes and crisis situations, and the most stressful event was exposure to a battered child [4]. However, this approach oversimplifies officer interactions with the public because the same event could end in many different ways. Even within a single classification of calls (e.g.*,* traffic accidents), a number of officer-, civilian-, supervisor-, and environment-level factors likely converge to effect how stressful that call is for an officer. An understanding of how these external factors impact an officer’s stress is important for researchers to consider when developing programs and interventions based upon the level of predicted stress that is likely to occur during the event.

Continuous exposure to stress and its subsequent impact on adverse events (e.g. injury; use-of-force; officer involved shooting) has also not been explored. In the United States, LEOs assigned to patrol functions respond to 911 calls for service and are typically dispatched to 8–10 calls per 8-h shift. Officers can be assigned to any call for service by the 911 operators and dispatchers, regardless of previous exposure to high-stress calls. We hypothesize that LEOs become susceptible to adverse events after repeated exposure to high-stress calls for service and ongoing exposures to stress continue throughout the shift.

The purpose of this study is two-fold:

1. Describe multi-level characteristics that define high-stress calls for service; and
2. Characterize factors that impact cumulative stress;

Guided by the dynamic recursive model of injury, which considers the implication of repeated exposure to high-risk events [15, 16], we hypothesize that intrinsic and extrinsic factors work together to create situations that put officers at higher risk for adverse events, like unintentional and intentional injury. We also hypothesize that repeated exposure to stressors over time acts as a catalyst in which adverse events are more likely to occur. We expect that the themes identified in our data will shed light on future testable intervention strategies to reduce stressors tailored for LEO’s and thereby address risk of adverse events.

## Methods

### Setting

The State of Texas has 150 law enforcement agencies, 61 of which cover the 2600 mile^2^ Dallas-Fort Worth geographic area. For the current study, we conducted a convenience sample from across three Dallas-Fort Worth area law enforcement agencies: 1) large urban with over 3000 officers; 2) suburban department with 400 officers; and, 2) rural department with 54 sworn officers.

### Data collection

The goal of data collection for this project was twofold: (1) to systematically identify stressors among LEOs, and (2) to engage law enforcement stakeholders and gather insights that may be used to identify gaps and opportunities to prevent adverse events in future policies and interventions. For this study, adverse event was defined as use-of-force, officer or civilian injury, civilian complaints, and discharge of a weapon.

A total of 28 patrol officers were recruited to participate in 8 focus groups (5 among urban; 2 among suburban; 1 among rural). At the suburban and urban departments, patrol officers meet for a briefing in the first 30 min of their shift. The purpose of these briefings is to take attendance, brief the officers on crime clusters and criminals in the area, and to describe unfinished business from previous shifts and administrative matters. Author KKJ attended details at patrol substations before each shift to recruit officers for focus groups. The recruitment briefing included a description of the project and clearly articulated that participation was voluntary and choosing not to participate would not impact employment. Officers interested in participating were instructed to sign-up on a paper-sheet by providing contact information either at the briefing or by email to author KKJ. Author KKJ scheduled all focus groups 1 week after recruitment and recruitment ended once results reached saturation. For the rural department, author KKJ sent an email to all eligible police officers explaining the study, explaining the procedures for participation, and instructed officers, that were interested, to respond via email of their interest.

Focus groups were conducted on-site at patrol substations, in a private conference room, 2 hours before officers’ shifts. Author KKJ conducted 8 focus groups from April 2019–February 2020. Before the focus group began, officers completed a brief, 8-item demographic survey. The semi-structured focus groups lasted approximately 1.5 h and began with a general discussion of the project. During the focus groups, LEOs were asked to identify stressors associated with the law enforcement profession. LEOs were also asked to describe their experiences and frequency of responding to high-stress calls for service and its broader effects on officer injury and health. Further, officers were asked to discuss how repeated stress is related to officer tenure, attitudes toward consecutive high-stress calls, confidence in how calls for service are correctly categorized by dispatch, decompression techniques used during and after a shift, and healthcare utilization to address health effects of stressors. Data collection for this study was approved by the Center for the Protection of Human Subjects at the University of Texas Health Science Center at Houston (HSC-SPH-18-0782). 

### Analysis

All focus groups were audio recorded and professionally transcribed. A multi-disciplinary team used a four-step approach to analyze qualitative data [17]. First, the research team collectively read transcripts collected from each focus group to develop a deeper understanding of the group discussion. Through this process, a deductive codebook was created to label text. We used these codes in group analysis sessions until we reached stability. Second, text was coded by the research team. We grouped emerging findings into categories of themes using an immersion-crystallization approach [18], which included inductive thematic identification. Third, transcripts were read by a second coder and coding inconsistencies were discussed and resolved by consensus. Finally, we considered how our findings relate to the literature. During this phase of analysis, dimensions connecting to the injury prevention recursive model of etiology emerged and we conducted an in-depth comparative analysis to understand differences. Nvivo version 12.0 software (QSR International Pty Ltd.) was used for all coding, organization, and data reduction.

## Results

Table 1 displays the demographics of study participants. Briefly, 86% were male, 57% were non-Hispanic White, and 54% were a college graduate. More than half of officers were married and 11% had a history of military service. The average tenure was 12 years, ranging from 1 to 34 years.

**Table 1:** Characteristics of Police Participant Population (*n* = 28)

	**%**	
Gender		
Male	86	
Female	14	
Race/Ethnicity		
Non-Hispanic White	57	
Hispanic	18	
Non-Hispanic Black	25	
Education		
High School Graduate/GED	11	
Some College or Technical School	32	
College Graduate	54	
Masters Graduate or Higher	4	
Marital Status		
Married	64	
Divorced	11	
Separated	4	
Never Married	14	
A Member of an Unmarried Couple	7	
Military Service	11	
	**Mean (SD)**	**Minimum, Maximum**
Age (years)	36 (10)	23, 61
Tenure (years)	12 (10)	1, 34

### Predisposing multi-level characteristics

There were five levels of themes that influenced officer stress: 1) officer characteristics (tenure, regularly riding with a partner, military experience, gender); 2) civilian behavior (resistance, displaying a weapon, behavior indicative of a mental health problem); 3) supervisor factors (micromanagement); 4) environmental factors (weather, time of day, time of year); and, 5) situational factors (audience present, call-types, complexity of situations). Evidence of each theme are displayed in Table 2.

**Table 2:** Multi-level evidence of themes reflecting high-stress calls for service for police officers

Theme	Evidence	Code Frequencies N(%)
**Officer-**		
Tenure	“When you’re a rookie, you got a high head, you want to prove yourself, want to be a hero so you do a lot of things just to try to prove yourself. Now everybody’s just like, okay, you’ve been on the job for at least ten years. You just wanna answer the call and go home.” “It takes a lot of experience, because a rookie just wanna go, go, go, go, go. “Oh, I answered 15 calls last night.” What do that mean? *[Laughs]* You know? So you just take your time. Just only one call at a time”	30 (3)
Partner	“You don’t want them to show up on your call but you’re with the same people all the time so you know who’s who, pretty much, so before you even get there, you have an assessment of what I’m gonna be doing or whether I need to watch my back or not.”	40 (3)
Military experience	“I come from a military background, as well, so a lot of this applies over, as well. How we react is based on our life experience”	13 (1)
Gender	“Why did you have to go [to the bathroom] all the way back to the station? You were like 15 min away. ‘I’m like, “Ah … Like if you really want to know I’m going to tell you and then you’re going to be sad.” “My partner for the longest time she was my classmate and the stories she would tell like stalking, hit-up all the time.” “When I was pregnant, the department didn’t have a policy in place for women to work behind the desk. I had to claim I was “unfit for duty” if I didn’t want to go out on the streets. Even with that, I could only claim to be “unfit for duty” for 6 months before I lost my job, so basically I was forced to answer calls until my second trimester. That was incredibly stressful.”	17 (1)
**Civilian-**		
Resistance	“Gonna be resistance, where you’re going to have to take action as far as physical action against a person or protect yourself or your partner from something physical happening when you can see it coming”	17 (2)
Weapon	“Any gun type of call. Anything with the potential for weapons or unknowns” “Why are you arresting him? It’s just a small gun.” And you’re looking at him like, “Really?” I mean that’s an actual quote, “It’s just a small gun why are you taking him to jail?”	23 (2)
Mental health	“Mental health, – I mean, sometimes it all pans out, but when you’re going there you’re like – ‘cause if you get into a fight, then everybody pulls their cameras out on their phones and it’s like “Well look, we’re trying to get him under arrest to take him to get some help” you know, but sometimes it works swimmingly and sometimes it doesn’t.”	45 (3)
**Supervisor-**		
Micromanagement	“You have to second guess everything because the management here, the internal affairs, the management in general; they don’t care about you, and they will make an example out of you, and they’ll hammer you. So you have to second guess everything because of upstairs.” “We have an official 40 min rule. So you have to be on a call 40 min or less. If you’re over 40 min, the sergeant call you and say “Why you on this call over 40 min?”. So you have to explain why you need a breather. Or you don’t explain and go to the next call stressed.” “Just sits there and watches GPS, watching what everybody’s doing, where everybody is, and just micromanaging the hell out of you”	65 (7)
**Environmental-**		
Weather	“The weather kind of dictates stress, too. If it’s cold out, you’re gonna ride clear for a little while. If it’s hot, you probably won’t get to clear but a few seconds, maybe a minute or two and you go into another call.” “During the wintertime is we call our downtime every year ‘cause it’s cold. People aren’t out. Your call load goes down. So yeah, you have a lot of low time where you can actually get a 50, but when it gets warm and summer hits, especially, you’re lucky if you can go take a pee break.”	10 (1)
Time of day/year	“Like just being able to see what’s going on better reduces your stress already, because you can see. In the dark you can’t. Like you’re walking up to a house, but like you literally cannot see if someone’s like hiding on the porch pointing a gun at you.” “2:00 to 3:00 A, roughly, ‘cause all the drunks get out at 2:00, so probably about 3:00 is when it dies down, and then after that, there’s usually a lull, unless we’re still playing catch up.”:“Summertime, we’ll be copping all night long. Days will be catching up our calls.” “Friday night, Saturday night, weekends, holidays, the Fourth of July, New Year’s Eve. Sports. Any time the Cowboys are playing. Any time they lose. Domestics go up every time the Cowboys lose. Calls go up in general every time the Cowboys play, but more calls come when they lose.”	34 (1)
**Situational-**		
Audience	“My focus is taken away from my job [when] having an audience. It would take away from my natural inclinations, how I would actually normally handle the call or handle the job because I’m thinking about who’s recording me, what I’m doing.” “I’ve seen them when there’s 20–30 people there and it’s not stressful to me. But then if you go to a family disturbance call everybody is calm except for one jackass and it’s like that makes everything stressful, because then you suspect this one person getting excited it’s going to excite the other 30 who are calm as we walk down. He’s upset at least but not crazy, crazy like this one guy. So that’s what’s stressful to me.” “And there’s a lot of people, and they’re all trying to tell you, “Hey, what are you doing?” And they’re all screaming, and this, and that, and that usually gets a little higher stress for me”	24 (3)
Call-types	“Rape, killings, stabbing, children abuse, women slapped,” “For me, I would probably have to say good domestic violence. When I say good, I mean really serious domestic violence calls where, like you said, you don’t know what you’re walking into because everybody’s so angry and tense and you’re waiting for the violence to turn on you, from the victim too. Sometimes that can happen too. Sometimes you go to arrest the suspect, well, the woman’s only means of income sometimes is getting ready to walk out the door. Well, she panics and then she turns on you. I’ve had that happen and that’s very stressful” “So my most stressful call was when a eight-year-old hung himself. I didn’t even know that eight-year-olds had that in their mind to do that kinda stuff.” “The highest-stress calls are the assist, because you know something’s going down then if somebody is asking for cover. Especially somebody that doesn’t ask for cover much. If they get on there and they yell that they never cover, Code Three, that is, that’s the worst right there.”	31 (3)
Complexity of situations	“Everyone wants to police the police, nowadays, so when you’ve got all of these elements that are happening at once, I think that’s what makes things kind of uneasy.” “It’s usually the totalitaria of the call, so it’s not any one specific call. It’s what elements, certain elements,”	21 (2)
Anticipation	“And then your partner says he has a bad feeling about this. I had the hair on the back of my neck stand up. Keep your Spidey senses to yourself, man.” “I’m gonna be the first one getting [to this call], so that’s a really high-stress thought process you’re going through as you’re going up to these calls or getting ready to go to that type of call, getting out of the car, getting a shield. We were the first ones going up. We were flanking the shield.” “I preach a lot on doing visualization exercises. So before the event even happens you’ve already seen it in your mind. So that when you actually get to it your mind is just like, Okay we’ve been here and we’ve handled this and this is what we’re going to do.”	42 (4)

### Cumulative stress

**Fig. 1:**
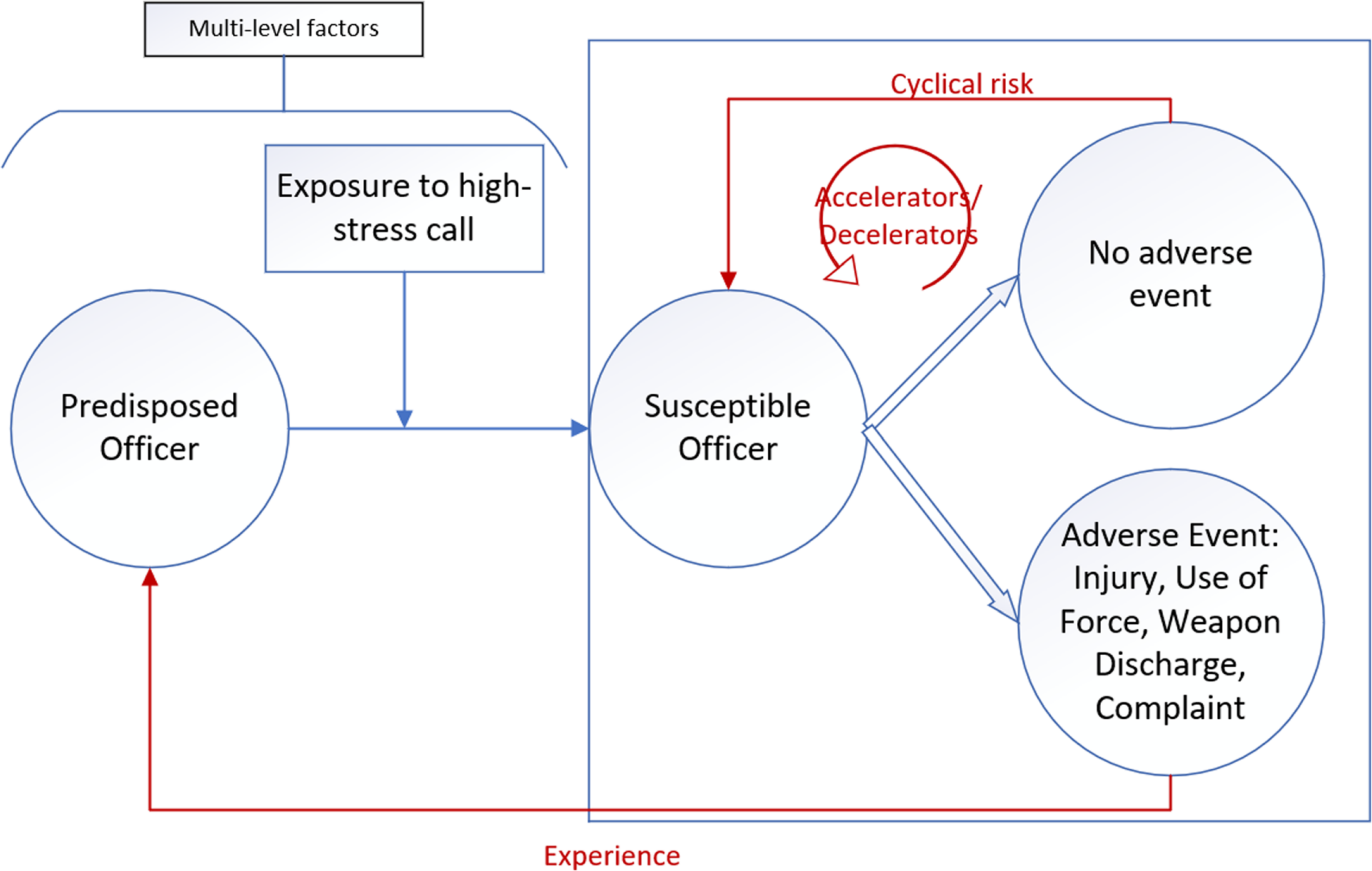
Impact of cumulative stress on law enforcement

We identified four themes that characterized cumulative stress: 1) cyclical risk; 2) accelerators; 3) decelerators; and 4) experience of an adverse event. The themes are denoted in red in Fig. 1 and described in further detail below.

#### **Cyclical risk**

After a high-stress call for service, officers did not “reset” their stress levels, but rather stress and susceptibility to adverse events was maintained through the remainder of the shift. Study participants described the experience of response to high-stress calls for service as a “*rollercoaster from hell*”. One officer reported that “*once you have the high-risk stress you don’t go back to like step one. It’s cumulative, so if nothing bad happens you’re kind of coming in right here again*”; “*What do you do after a high stress call? Go to the next one*”. In other words, officers perceived that the cyclical nature of responding to 911 calls caused prolonged high stress. One officer reported:

“*If the calls are just routine where it’s just easy. But when you do go to the one where your heart rate’s up and maybe you have to put your hands on somebody or something like that, then just coming right down from that and getting right back into the call answering and stuff is very aggravating and frustrating because you’ve gone from 0 to 100 like that, and then to come back down to 0 and get back in and just start answering routine calls can be very stressful*”.

Officers themselves recognize potential for a cyclical cycle pattern to increase the likelihood of adverse events, like use-of-force and/or officer and civilian injury. In other words,

“*That’s where things can get raw and can get messy on your next call, if you take that stuff from the previous call into as if it was something really impactful to you and then you go right to another call, that’s where you can potentially mess up and make something go wrong*”.

### Accelerators

Officers described two features, burnout and work performance, that accelerate the recursive stress cycle.

#### Burnout

Officers described burnout from jumping call to call. For example, officers stated:

“*There’s really dynamic calls and you’re going one dynamic call to another dynamic call, and your stress level goes up, and then your fuse gets real short*”; “*It was [lights and sirens] call after [lights and sirens] call after [lights and sirens] call, and I was tired. I was tired. We still had three or four hours left on shift*”; and “*You go to a high risk call and your heart rate goes up and your adrenaline starts to pump and then nothing happens and you do it again, and again, and again, it eventually has a lingering effect on your body and I remember it just drains me*”.

Officers reported that the burnout can then lead to adverse events even quicker. One officer reported: “*It’s the burnout and the other effects that come with jumping call, to call, to call that’ll make it where you screw up*”. Another officer said:

“*I think where the bad part comes in is when you get the burnout and that’s when the mistakes happen or you do something wrong or something bad happens to you because you’re burnt out or you’re so stressed out to the point where you’re not paying attention like you normally would do.*”

#### Pressure to move forward

Officers consistently reported pressure to move to the next call, even if they weren’t mentally ready. For example, one officer reported that his supervisor asked him/her, “*Are you done with your call, wink, wink, hint, hint? Cause there’s a call coming out and you’re riding some [unimportant call] and everybody knows you’re riding some [unimportant call]. Go ahead and [go to the next call]*”.

### Decelerators

During focus groups, officers identified three specific compensating, and related, behaviors that map to decelerators of cyclical stress: 1) taking a break; 2) changing mental state; and, 3) addressing mental health over time.

#### Take a break

Officers reported that “*sometimes you’re just not ready*” for the next call. “*You’re already amped up. So when I saw that, that’s why I told myself that ‘You know what? No’. If I need to pull over for five minutes, then I’m just gonna do it.*” Specifically, patrol officers have found “*to work the system to our favor because you can’t just keep going on calls, especially when there’s 300, 400 calls sitting in. It’s just call, to call, to call.*” One officer reported: “*I’ve learned now you’ve just gotta take your break. If you get on a high stress call, it could be three or more [high priority calls] holding. I’m still gonna take a little break, go to 7-11, chill out for a little bit, get my mind right; then I go answer for another call. I just don’t clear right away, boom, boom, take another call.*”

#### Change mental state

Officers found that changing their mental state on the way to the next call helped to attenuate the probability of an adverse event. One officer reported:

“*Whenever I leave one call and I’m heading to the next call, by the time I get to the next call, I’ve kinda zoned out of the call before, and I get to the next call and you can even ask me ‘Hey what happed at the last call?’ … I don’t remember.*”

A field training officer said: “*Like I tell my rookies, if you make a mistake, leave it with that call back there. Focus on your call now because if you focus on that mistake, you’re gonna mess up on your future calls*”.

#### Addressing mental health over time

Officers also mentioned the importance of addressing their own mental health throughout their careers. One officer described:

“*When you get in that [stress] cycle, if we are already ahead of our mental health, of our decompression and stuff, it’ll make all this stuff more worth it and easier to handle. If we feel like we’re taken care of, if we feel like we can decompress well, and we can tackle our mental health well, we can handle [high stress] because I’m okay up [in my head]*.”

Another officer mentioned: “*If you’re not accepting it, you’re just like sitting there like not really talking or not really dealing with whatever you’re going through, and you just bottle it up then obviously you know that doesn’t help”*.

### Experience of an adverse event

Once an officer experienced an adverse event, their tolerance for high-stress became a protective intrinsic factor for future calls. For example, one officer stated, “*I mean I’ve never gotten that pumped or anything again. And that’s why I don’t get- I really don’t think- I get stressed out as much anymore, because you’ve hit that level. You have that new threshold of stress*”; “*Once you’ve hit this adrenaline that’s the highest it will ever go, you’ll never hit it again*”; “*You know you put it all on that scale and you’ll never hit ten again. It’s like, ‘What else you got for me?’*”

Many officers reported that tenure influenced this phenomenon:

“*the stress doesn’t come as often because you’ve done everything a hundred times, by the time you get to seven, eight, nine, ten years*”;

“*Especially I would say younger officers they have an even harder time bringing it back down. Like if you’ve been on a while you’re like okay, you shake it off a little bit better and you’re like Okay let’s go to the next one. It’s over. We are fine, we made it. But a lot of the younger officers or even ones that already have [post-traumatic stress disorder] they kind of stay stuck in the loop at lot longer or they go up a couple steps instead of one I would say.*”

## Discussion

Results from this study suggest that intrinsic (i.e. officer characteristics) and extrinsic (i.e. situational, environmental, civilian, and supervisor) factors converged to produce stress in law enforcement officers, and thus increase the likelihood of adverse event occurrence. Our findings highlight how stress comes from multiple levels of the organization. This is the first study to qualitatively examine how officers from urban, suburban, and rural law enforcement agencies characterize the stressors that patrol officers face on a daily basis. Results from this study are consistent with past quantitative studies, which have demonstrated that multi-level factors influence LEO-civilian interactions that result in adverse events, like use of force [19, 20] escalation of force [21] and injury [19]. In this study, it is notable that we found only civilian behavior was reported as a contributing influence for officer stress; not civilian personal factors, like civilian appearance (e.g. race/ethnicity; tattoos; body mass index). Our previous studies of adverse events have also supported this finding that indicators of civilian behavior (e.g.*,* presence of a firearm at the scene, civilian displayed active aggression, substance impairment or displaying symptoms of mental illness) were more robust predictors of adverse events than civilian appearance [19]. Clearly, while officer-civilian interactions are complex and likely affected at multiple levels, civilian behavioral indicators are routinely documented and may be more objectively actionable through system-level interventions.

Continuous exposure to stress and risk for adverse events (e.g. injury) is not unique to police officers. The framework used in this study to identify accelerators and decelerators of cumulative stress was originally devised by Meuwisse and colleagues, with the goal of using their injury prevention recursive model to inform primary, secondary, and tertiary prevention strategies for sports injury [15, 16]. The injury prevention recursive model considered the implications of repeated exposure and whether such exposure predicts injury or recovery from injury. Results from the present study suggest that officers’ experiences fit the constructs of the recursive model of injury. Officers identified several accelerators of cumulative stress, including burnout and pressure to respond to subsequent calls for service. Multiple decelerators were also mentioned; interestingly, some were nominated in a stepwise fashion: taking a break; change mental state before next call; and, seek care for their own mental health. These indigenous practices suggest that an intervention is possible because officers are aware of the occupational hazards and stressors they experience in the course of their occupation.

Themes identified from our data provide several opportunities for testing future interventions tailored for LEOs that target those multi-level factors that accelerate or decelerate the effects of cumulative, cyclical stress. First, front-line patrol supervisors may be educated on effective leadership strategies to simultaneously address occupational demands, such as increasing officer productivity and lowering response times, while balancing officer needs at the individual level, including stress levels. Past literature has shown that leadership-generated stressors substantially impact officers’ mental health and effectiveness in doing their jobs [22]. In other occupations, it has also been shown that when workers are less stressed, their productivity and job satisfaction incrementally increase [23–25]. Second, evidence-based solutions used in other occupations, like mindfulness or mediation, can be tested to see if such strategies successfully manage anticipatory stress when responding to calls for service and the aftermath of high-stress calls for service. For example, short breathing exercises might be routinely incorporated into existing time used for drafting a report after officers respond to a high-stress call for service. Third, our findings emphasize the chronic mental health needs of LEOs to be evaluated. This would start with estimating the prevalence of undiagnosed mental health problems among LEO’s and assessing how current services do and do not address this unique population. Additional research is critically needed in all these areas to test the feasibility, acceptability, and effectiveness of reducing stress, and thus reducing adverse events between officers and the communities they serve.

A final implication of our study of multi-level factors highlights the need for systemic approaches that could break the cycle of stress among LEOS. Thus, recognizing that calls are the primary route by which LEOs interact with civilians, one possible systems approach might be to leverage technological advances to intervene on the management of calls. The computer-aided dispatch (CAD) system could be devised to equip officers with the knowledge of the complexity of calls by, for example, triangulating information such as time of day, type of call, and responding officer characteristics, to create a stress continuum scale. When the scale is highest, the CAD system would then inform the officer in route to the call. Furthermore, CAD could be programmed to interrupt this repetitive, cumulative process by restricting the number of consecutive high-intensity calls to which an officer is permitted to respond.

### Limitations and strengths

This study should be considered in light of several limitations and strengths. First, because officers were recruited from a single geographic area, Dallas-Fort Worth metroplex, the generalizability is limited. However, we did recruit from multiple departments that serve urban, suburban and rural communities; this breadth is rare in this field of research and strengthens external validity. Moreover, the demographic accrual of our study sample does represent the demographic composition of the participating departments. Second, participants for our study were recruited using convenience sampling; as a result, officers who self-selected to participate might be more forthcoming or have unique experiences related to stress compared to the general population of officers. In light of these limitations, our study was the first to gather qualitative data on the cumulative stressors experienced by patrol officers theoretically guided by the injury prevention recursive model.

## Conclusion

In conclusion, the intensity of and stress associated with call response that is experienced by law enforcement officers on a day-to-day basis could potentially explain adverse events, like officers’ high rates of injury. Our long-term goal is to develop and test a number of strategies that address officer stress, like leveraging technological advances, and to rigorously evaluate the effectiveness of these interventions on police and civilian injury, use-of-force, civilian complaints, and law enforcement mental health.

## Data Availability

The datasets used and/or analyzed during the current study are available from the corresponding author on reasonable request.

## Abbreviations

LEO: Law enforcement officer
CAD: Computer-assisted dispatch
PTSD: Post-traumatic stress disorder
